# SOCS3–microtubule interaction *via* CLIP-170 and CLASP2 is critical for modulation of endothelial inflammation and lung injury

**DOI:** 10.1074/jbc.RA120.014232

**Published:** 2021-01-09

**Authors:** Pratap Karki, Yunbo Ke, Chen-Ou Zhang, Yue Li, Yufeng Tian, Sophia Son, Akihiko Yoshimura, Kozo Kaibuchi, Konstantin G. Birukov, Anna A. Birukova

**Affiliations:** 1 Department of Medicine, University of Maryland School of Medicine, Baltimore, Maryland, USA; 2 Department of Anesthesiology, University of Maryland School of Medicine, Baltimore, Maryland, USA; 3 Section of Pulmonary and Critical Care Medicine, Department of Medicine, University of Chicago, Chicago, Illinois, USA; 4 Department of Microbiology and Immunology, Keio University, Tokyo, Japan; 5 Department of Cell Pharmacology, Nagoya University, Nagoya, Japan

**Keywords:** endothelial dysfunction, lung injury, inflammation, permeability, microtubule, cytoskeleton, cell junction, SOCS3, *Staphylococcus aureus*, ALI, acute lung injury, ANOVA, analysis of variance, BAL, bronchoalveolar lavage, EC, endothelial cell, HKSA, heat-killed *Staphylococcus aureus*, HPAEC, human pulmonary artery endothelial cell, ICAM-1, intercellular adhesion molecule-1, IL-6, interleukin-6, JAK-STAT, Janus kinase-signal transducer and activator of transcription, KIR, kinase inhibitory region, MT, microtubule, MTOC, microtubule organizing center, NS, nonspecific, SOCS, suppressors of cytokine signaling, TER, transendothelial electrical resistance, VCAM-1, vascular cell adhesion molecule-1, WT, wild-type

## Abstract

Proinflammatory cytokines such as IL-6 induce endothelial cell (EC) barrier disruption and trigger an inflammatory response in part by activating the Janus kinase-signal transducer and activator of transcription (JAK-STAT) pathway. The protein suppressor of cytokine signaling-3 (SOCS3) is a negative regulator of JAK-STAT, but its role in modulation of lung EC barrier dysfunction caused by bacterial pathogens has not been investigated. Using human lung ECs and EC-specific SOCS3 knockout mice, we tested the hypothesis that SOCS3 confers microtubule (MT)-mediated protection against endothelial dysfunction. SOCS3 knockdown in cultured ECs or EC-specific SOCS3 knockout in mice resulted in exacerbated lung injury characterized by increased permeability and inflammation in response to IL-6 or heat-killed *Staphylococcus aureus* (HKSA). Ectopic expression of SOCS3 attenuated HKSA-induced EC dysfunction, and this effect required assembled MTs. SOCS3 was enriched in the MT fractions, and treatment with HKSA disrupted SOCS3–MT association. We discovered that—in addition to its known partners gp130 and JAK2—SOCS3 interacts with MT plus-end binding proteins CLIP-170 and CLASP2 *via* its N-terminal domain. The resulting SOCS3–CLIP-170/CLASP2 complex was essential for maximal SOCS3 anti-inflammatory effects. Both IL-6 and HKSA promoted MT disassembly and disrupted SOCS3 interaction with CLIP-170 and CLASP2. Moreover, knockdown of CLIP-170 or CLASP2 impaired SOCS3–JAK2 interaction and abolished the anti-inflammatory effects of SOCS3. Together, these findings demonstrate for the first time an interaction between SOCS3 and CLIP-170/CLASP2 and reveal that this interaction is essential to the protective effects of SOCS3 in lung endothelium.

Cytokine-derived signaling plays an essential role in modulating all kinds of host immune responses. Thus, a precise regulatory mechanism controlling the duration and magnitude of cytokine-activated inflammatory signaling is vital for body defense against infectious pathogens as well as for preventing hyperactive innate immune response described in several immunopathologies (1). Suppressors of cytokine signaling (SOCS) provide such regulatory checkpoints to inhibit cytokine signaling by acting as a canonical negative feedback loop (2, 3, 4, 5). Janus kinase-signal transducer and activator of transcription (JAK-STAT) pathway is the principal signaling axis employed by many cytokines including interleukins and interferons (6). The binding of cytokines to their receptors activates JAK by its cross-phosphorylation; the active JAK then phosphorylates tyrosine residues in cytokines receptors. It leads to the recruitment and phosphorylation of STATs, which undergo nuclear translocation and stimulate the transcription of cytokine responsive genes including SOCS (5). Out of eight SOCS members, SOCS1 and SOCS3 are potent inhibitors of JAK-STAT pathway with their ability to directly interact with JAK (2, 4). A critical physiological importance of SOCS3 is highlighted by the findings that SOCS3 knockout mice die perinatally due to defective placenta formation and erythrocytosis (7, 8). Furthermore, with its profound anti-inflammatory roles in cytokines and growth factors signaling, SOCS3 has been suggested to be a potential therapeutic target for lung inflammation, autoimmune diseases, and metabolic disorders (9, 10, 11, 12).

In resemblance to other SOCS family members, SOCS3 also contains a central SRC homology 2 (SH2) domain and a C-terminal SOCS box (2, 5). Additionally, SOCS3 has a kinase inhibitory region (KIR) at the N-terminal adjacent to SH2 domain as in SOCS1 (13). KIR is thought to act as a pseudo-substrate for JAK and thus efficiently inhibits its tyrosine kinase activity (14, 15). SOCS3 also binds to gp130, an interleukin-6 (IL-6) family receptor subunit, owing to the inhibition of signaling induced by them (16). IL-6 is a proinflammatory cytokine with its major role in various inflammatory diseases (17); binding of SOCS3 to IL-6 receptor gp130 at Tyr757 represses IL-6-induced inflammation (16). Besides inhibiting JAK activation through direct interaction, SOCS box present in SOCS3 associates with elongin proteins that are a part of ubiquitin-mediated proteasomal degradation (18, 19). The importance of SOCS box of SOCS3 in interfering cytokines signaling was evident with the hyperresponsiveness of mice lacking SOCS box to granulocyte-colony stimulation factor (20).

Endothelial cells (ECs) respond to various inflammatory challenges by release of proinflammatory cytokines (21). In turn, these secreted cytokines induce EC barrier disruption and increase the expression of EC adhesion molecules: intercellular adhesion molecule-1 (ICAM-1), vascular cell adhesion molecule-1 (VCAM-1), and E-selectin that contribute to the recruitment of neutrophils to the endothelium, ultimately leading to inflammation (22). Increased endothelial permeability and vascular inflammation are a common feature of many highly morbid conditions including sepsis, acute lung injury (ALI), and acute respiratory syndrome (23, 24). A potential role of SOCS3 as a major negative regulator of cytokine signaling in inflammatory agonist-induced endothelial dysfunction warrants further investigation.

Microtubules (MTs) are now considered as a major player in the regulation of EC barrier function (25). Our previous studies have shown that MT destabilization may be caused by inflammatory agonists and leads to endothelial barrier dysfunction and further promotes inflammation (26, 27). Interestingly, SOCS3 expressed in myeloid cell line has been shown to interact with MT-associated protein MAP1S, but SOCS3 only partially colocalized with perinuclear microtubule organizing center (MTOC) in HeLa cells overexpressing MAP1S (28). MT destabilization by nocodazole prolonged IL-6 inflammatory signaling (29), although molecular partners regulating SOCS3 activity remained unclear. Thus, better understanding the role of MT in regulation of SOCS3 function in the settings of pathogen-induced endothelial permeability and inflammation is of high importance for better understanding of protective feedback mechanisms in various models of lung injury (30, 31, 32).

In the study presented here, we used gain or loss-of-function molecular approaches and EC-specific SOCS3 knockout mice to investigate the role of SOCS3 in restraining inflammatory agonist-induced endothelial permeability and inflammation. We also investigated the potential mechanisms and involvement of MT dynamics in regulating these pathological cascades.

## Results

### SOCS3 knockdown augments EC permeability and inflammatory responses

**Figure 1:**
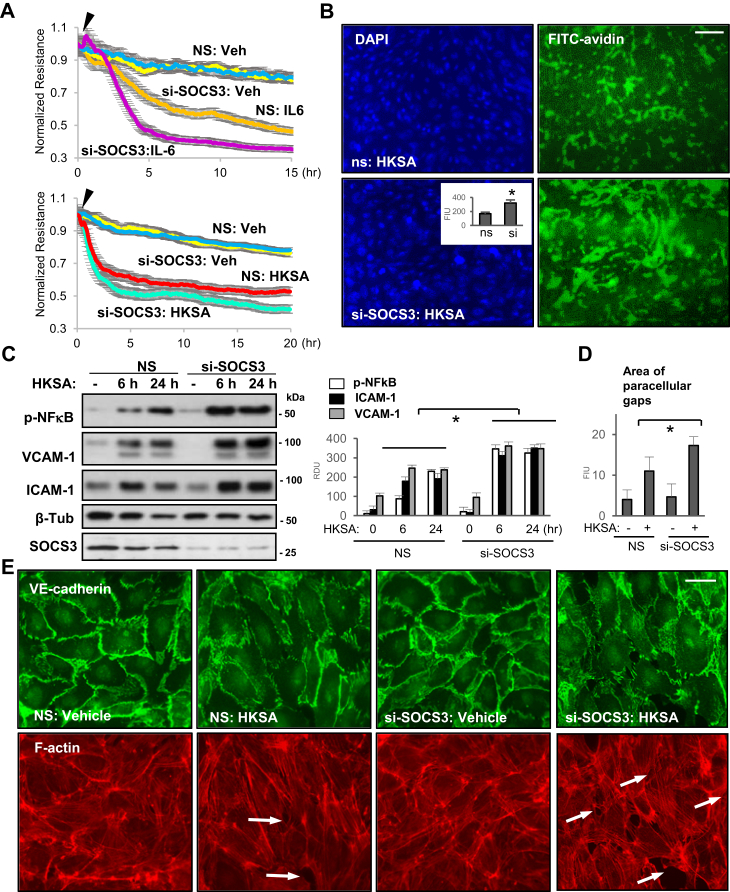
**SOCS3 knockdown augments IL-6 or HKSA-induced endothelial permeability and inflammation.** HPAECs transfected with control nonspecific (NS) or SOCS3-specific siRNA (si-SOCS3) were stimulated with vehicle (Veh), IL-6 (40 ng/ml) or HKSA (5 × 10^8^ particles/ml). *A*, TER was monitored for indicated time periods. *B*, HPAECs on plates coated with biotinylated gelatin were transfected with NS or si-SOCS3 followed by HKSA treatment, 6 h. XPerT assay was performed as described in Experimental procedures; FITC-avidin bound to gelatin-coated base was visualized by immunofluorescence microscopy. DAPI counterstaining shows cell nuclei. Bar: 50 μm. *Inset*: imaging analysis of FITC-avidin fluorescence signal; n = 4, ∗*p* < 0.05. *C*, western blot (WB) analysis of phospho-NFκB, VCAM-1, and ICAM-1 levels in control and HKSA-stimulated HPAEC. Membrane reprobing with SOCS3 antibody was used to verify siRNA-mediated knockdown; β-tubulin served as a loading control. The bar graph depicts the quantitative densitometry of western blots; n = 5, ∗*p* < 0.05. *D* and *E*, VE-cadherin immunostaining was performed visualize adherens junctions; F-actin was stained using Texas Red phalloidin. Gaps in cell monolayers are marked by arrows. Bar: 10 μm. Bar graphs depict quantitative analysis of intercellular gaps (*D*); n = 4, 10 microscopic fields per condition; ∗*p* < 0.05. FIU, fluorescence intensity units; RDU, relative density units.

SOCS3 is a known negative regulator of inflammation (10), but its role in modulation of inflammatory lung endothelial barrier dysfunction is not known. To define the role of SOCS3 in modulating IL-6 and HKSA-induced permeability, we measured transendothelial electrical resistance (TER) in EC transfected with nonspecific (NS) or SOCS3 targeting siRNA. The results showed that depletion of endogenous SOCS3 markedly increases IL-6- or HKSA-induced endothelial barrier disruption (Fig. 1*A*). In parallel, we also examined endothelial permeability to macromolecules using XPerT assay described in Methods. HKSA-induced endothelial permeability evidenced by increased FITC fluorescence intensity was much greater in HKSA-treated EC transfected with SOCS3 siRNA (Fig. 1*B*). HKSA-induced inflammatory responses in EC are mediated by activation of the NF-κB pathway and upregulation of EC adhesion molecules VCAM-1 and ICAM-1 (32). To investigate the role of SOCS3 in regulating these HKSA-activated inflammatory cascades, we determined the protein levels of phospho-NFκB, VCAM-1, and ICAM-1 in SOCS3-depleted EC. We observed a significant increase of all three EC inflammatory marker proteins following HKSA challenge of SOCS3 siRNA-transfected cells, as compared with NS siRNA-treated groups (Fig. 1*C*). Immunofluorescence analysis of HKSA-induced disruption of EC junctions showed a more pronounced discontinuous VE-cadherin staining in EC with SOCS3 knockdown reflecting more severe endothelial barrier disruption (Fig. 1DE). Consistently, actin staining with Texas Red Phalloidin revealed increased formation of paracellular gaps in HKSA-challenged EC with SOCS3 knockdown, as compared with NS siRNA-transfected controls (Fig. 1, *D* and *E*). Altogether, these data suggest a critical role of SOCS3 in negative regulation of HKSA- and IL-6-induced EC permeability and inflammation.

### EC-specific SOS3 knockout exacerbates HKSA or IL-6-induced lung injury in mice

**Figure 2:**
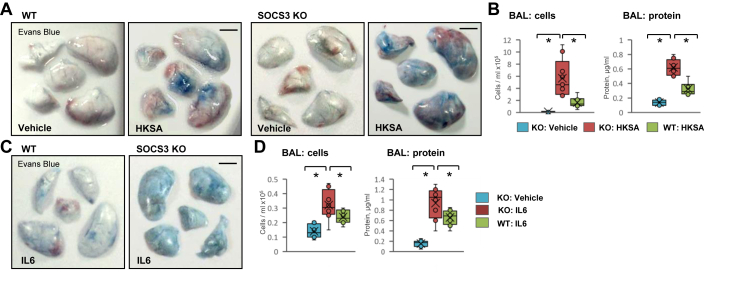
**EC-specific SOCS3 knockout exacerbates HKSA or IL-6-induced ALI**. *A*, wild-type (WT) or EC-specific SOCS3 knockout (SOCS3 KO) mice were injected with HKSA (intratracheally, 2 × 10^8^ bacterial cells/mouse), and after 18 h vascular leak was determined by Evans blue extravasation assay. Normal saline was used as a vehicle. Bar: 4 mm. *B*, the total cell count (left) and total protein content (right) were determined in bronchoalveolar lavage (BAL) fluid. Bar diagrams present individual data points, means (*cross*), and medians (*line*); n = 8, ∗*p* < 0.05. *C*, WT and SOCS3 KO mice were injected with IL-6 (i.v., 100 ng/mouse) and Evans blue extravasation assay was performed. Bar: 4 mm. *D*, cell count and protein content were measured in BAL; n = 6, ∗*p* < 0.05. The bar diagrams represent individual data points and averages with standard error.

The role of SOCS3 in protecting against inflammatory agonist-induced endothelial dysfunction was further validated *in vivo*. We induced ALI in wild-type (WT) and EC-specific SOCS3 KO mice by intratracheal instillation of HKSA or IL-6, as described in Methods and evaluated lung injury parameters. SOCS3 KO mice challenged with HKSA exhibited more pronounced accumulation of the Evans blue dye in the lung parenchyma, suggesting a higher degree of vascular leak in comparison with WT littermates (Fig. 2*A*). Analysis of bronchoalveolar lavage (BAL) samples showed an increase in the number of total cells and protein content in SOCS3 KO mice as compared with their matching controls (Fig. 2*B*). Likewise, IL-6 induced a more severe vascular leak in SOCS3 KO mice, as assessed by Evans blue extravasation assay (Fig. 2*C*). Consistently, a higher number of cells and increased protein content were observed in BAL from SOCS3 KO mice following IL-6 injection as compared with matching WT controls (Fig. 2*D*).

### SOCS3 overexpression suppresses inflammatory agonist-induced endothelial dysfunction

**Figure 3:**
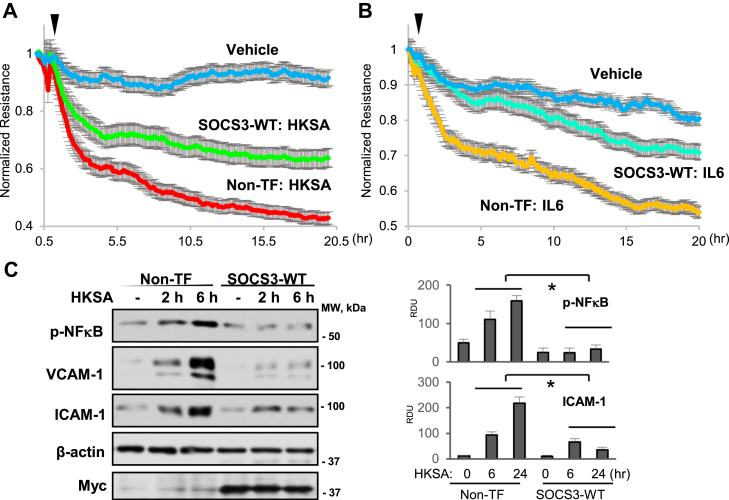
**SOCS3 overexpression attenuates HKSA or IL-6-induced endothelial barrier disruption and inflammation**. *A* and *B*, ECs were transfected with Myc-tagged SOCS3 or left nontransfected (Non-TF) followed by HKSA (*A*) or IL-6 (*B*) treatment. TER was monitored over 20 h. *C*, western blot analysis of phospho-NFκB, VCAM-1, and ICAM-1 levels in HKSA-treated EC with ectopic expression of Myc-SOCS3 and in nontransfected controls. Probing with β-actin was used as a loading control. Reprobing with Myc antibody was used to verify ectopic SOCS3 overexpression. Bar graphs depict analysis of western blotting data by quantitative densitometry; n = 5, ∗*p* < 0.05. RDU, relative density units.

To further determine the role of SOCS3 in protecting EC against inflammatory agents, we tested if overexpression of WT SOCS3 (SOCS3-WT) has any impact on HKSA- or IL-6-induced endothelial permeability and inflammation. TER analysis revealed that HKSA-induced EC permeability was attenuated in cells overexpressing SOCS3 as compared with nontransfected groups (Fig. 3*A*). Similar results were observed with IL-6 stimulation, as ectopic expression of SOCS3 markedly suppressed IL-6-induced endothelial barrier disruption (Fig. 3*B*). Next, we determined the protein levels of phospho-NFκB, VCAM-1, and ICAM-1 after HKSA challenge in control or SOCS3-transfected cells. The results demonstrated that SOCS3 overexpression diminished HKSA-induced upregulation of these proteins (Fig. 3*C*), suggesting a major anti-inflammatory role of SOCS3 in pulmonary EC.

### SOCS3 is enriched in the microtubule fraction and interacts with MT-associated proteins

**Figure 4:**
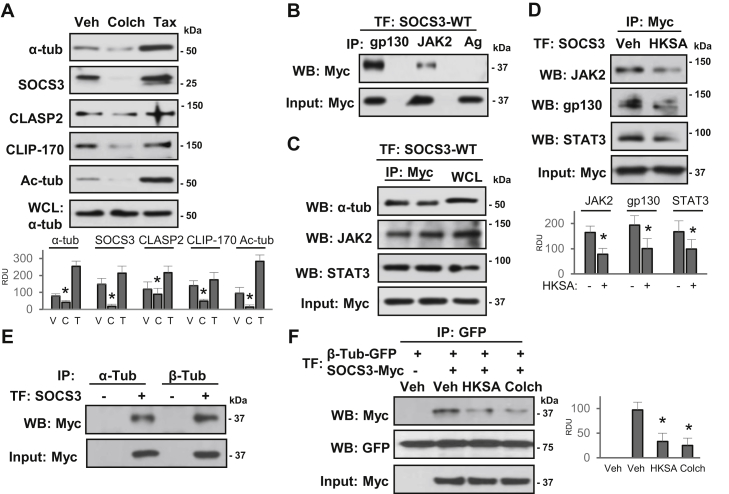
**SOCS3 associates with MT and inflammatory signaling molecules**. *A*, cells were treated with MT depolymerizing (Colch: Colchicine, 0.5 μM, 1 h) and polymerizing (Tax: Taxol, 0.5 μM, 30 min) agents followed by MT fractionation. SOCS3, CLIP-170, CLASP2, acetylated-tubulin (Ac-tub), and α-tubulin protein contents in MT-enriched fractions were determined by western blot. Probing of total cell lysates with α-tubulin antibody was used to ensure equal input. Bar graphs depict analysis of western blotting data after cell stimulations with vehicle (V), colchicine (C) and taxol (T); n = 5, ∗*p* < 0.05. *B*, ECs expressing Myc-tagged SOCS3 were used in co-IP assays with gp130 or JAK2 antibodies; incubation with protein G agarose (Ag) alone served as a negative control. SOCS3 in immunoprecipiates was detected by Myc antibody. Probing of total cell lysates with Myc antibody was used to verify equal IP inputs. *C*, cells overexpressing SOCS3-WT were used for co-IP with Myc antibody; immunocomplexes were probed with α-tubulin, JAK2, and STAT3 antibodies. Probing of total cell lysates with Myc antibody was used to verify equal IP inputs; TF: transfection. *D*, control and HKSA-stimulated (6 h) EC with Myc-SOCS3 ectopic expression were lysed, and co-IP assays with Myc antibody followed by probing for JAK2, gp130, and STAT3 were performed. Probing of total cell lysates with Myc antibody was used to verify equal inputs. *E*, association of SOCS3 with MT in EC expressing Myc-SOCS3 was assessed by co-IP with α- or β-tubulin antibodies followed by probing for Myc. *F*, ECs expressing GFP-β-tubulin or Myc-SOCS3 were stimulated with HKSA or colchicine followed by co-IP with GFP antibody. SOCS3 interaction with tubulin was detected by western blot with Myc antibody; membranes were reprobed for GFP to verify equal pulldown conditions. Equal volumes of total cell lysates were probed with Myc antibody to confirm SOCS3 ectopic expression. Bar graphs in panels (*A*, *D*, *F*) depict the results of quantitative densitometry of western blots; ∗*p* < 0.05, n = 5 for each experiment. RDU, relative density units.

Growing evidence suggests that MT-associated proteins play a critical role in stabilizing EC barrier integrity and controlling inflammation (25, 33). Thus, we next tested whether EC barrier protective effects of SOCS3 are MT-dependent. We performed MT fractionation assay and analyzed protein levels in MT-enriched fractions. As expected, MT stabilizer taxol increased, whereas MT depolymerizing agent colchicine decreased levels of polymerized tubulin (Fig. 4*A*). Likewise, levels of acetylated tubulin, reflecting the pool of stabilized MT, and MT plus-end binding proteins CLIP-170 and CLASP2 were increased upon taxol treatment and decreased after colchicine exposure. Importantly, the results showed an abundant presence of SOCS3 in the MT fractions, its increased content after taxol, and decreased SOCS3 level in colchicine-treated EC (Fig. 4*A*).

To gain insight into the mechanisms of SOCS3-mediated protection of endothelial function, we investigated the potential binding partners that might play a role in controlling EC permeability and inflammation. Initially, in our experimental settings we determined the interaction of SOCS3 with known inflammatory signaling proteins gp130 and JAK2. The overexpression of Myc-tagged SOCS3-WT followed by coimmunoprecipitation (co-IP) analysis demonstrated a strong association of SOCS3 with IL-6 receptor gp130 and JAK2 (Fig. 4*B*). This interaction was further confirmed by reverse coimmunoprecipitation, where Myc antibody was used for co-IP and immunocomplexes probed for tubulin, JAK2 and STAT3. The results showed that SOCS3 associates with tubulin, JAK2, and STAT3 in lung EC (Fig. 4*C*). More importantly, HKSA challenge of EC largely impaired the interaction of SOCS3 with JAK2, gp130, and STAT3 (Fig. 4*D*). The association of SOCS3 with MT was further confirmed by co-IP assays in cells overexpressing SOCS3. The results demonstrated that exogenously expressed SOCS3 robustly associated with α- or β-tubulin, structural units of the MT (Fig. 4*E*). Strong association of SOCS3 with the MT prompted us to test whether inflammatory agonists disrupt this interaction. Co-IP assays following DNA cotransfections showed that the interaction between SOCS3 and β-tubulin was markedly reduced by HKSA challenge, almost to a similar level caused by colchicine (Fig. 4*F*).

### Role of MT in SOCS3-regulated endothelial function

**Figure 5:**
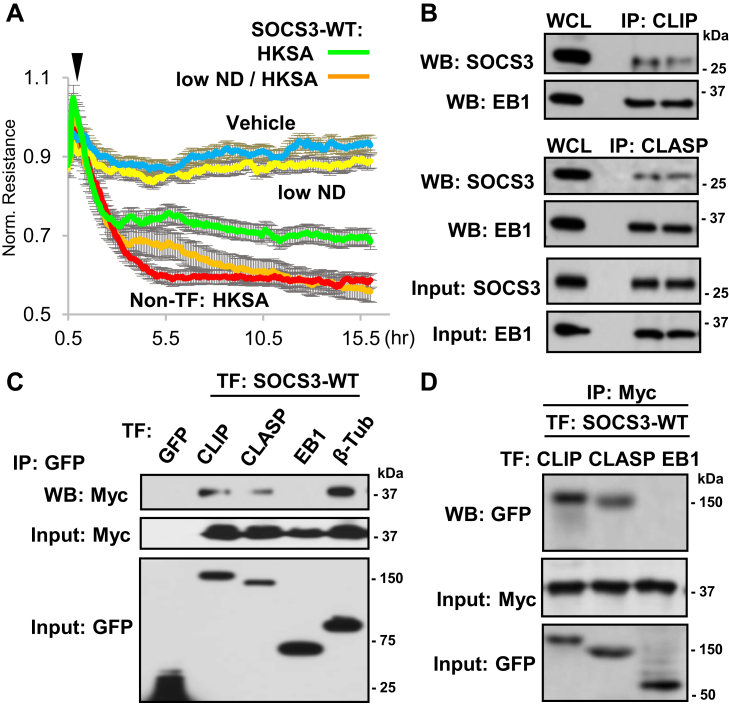
**SOCS3 interacts with MT plus-end-binding proteins**. *A*, ECs with ectopic expression of Myc-SOCS3 were treated with HKSA, with or without low-dose nocodazole (low ND, 0.05 nM); EC permeability was monitored by TER; n = 5. *B*, interaction of Myc-SOCS3 with MT-associated proteins analyzed by co-IP assay with CLIP-170 and CLASP2 antibodies and immunoblotting for Myc. EB1 was monitored as a known interacting partner of CLIP and CLASP. SOCS3 and EB1 contents in total cell lysates were monitored to verify equal protein amounts used for IP. Shown are representative results of four independent experiments. *C*, ECs cotransfected with GFP-tagged CLIP-170, CLASP2, EB1, or β-tubulin and Myc-SOCS3 were used for co-IP assays with GFP antibody and western blot detection of Myc-SOCS3. Myc-SOCS3 detection in total cell lysates was used to verify equal inputs. Shown are representative results of five independent experiments. *D*, co-IP of EC coexpressing GFP-tagged CLIP-170, CLASP2, or EB1 and Myc-SOCS3 using Myc antibody for co-IP followed by probing for GFP. Total cell lysates probed for Myc and GFP served as normalization controls. Shown are representative results of four independent experiments; TF: transfection.

To determine the functional significance of the MT-SOCS3 association, we assessed endothelial barrier integrity in the presence of low dose of nocodazole that blocks normal MT growth without causing global MT disassembly (34). Ectopic expression of SOCS3-WT attenuated HKSA-induced endothelial permeability, but this protective effect was impaired by EC treatment with nocodazole (Fig. 5*A*). These findings support the importance of MT integrity in maintaining SOCS3 function. We next investigated whether MT-associated proteins interact with SOCS3 in EC. Coimmunoprecipitation data demonstrated that SOCS3 was associated with CLIP-170 and CLASP2, but not with another MT plus-end binding protein, EB1 (Fig. 5*B*). In agreement with published studies, CLIP-170 and CLASP2 interacted with EB1, which served as a positive control to analysis of SOCS3 interaction with EB1. These interactions were further verified by cotransfection experiments where Myc-tagged SOCS3 and GFP-tagged CLIP-170, CLASP2, EB1, and β-tubulin were overexpressed. The co-IP assays with GFP antibody showed that SOCS3 interacts with CLIP-170, CLASP2, and tubulin, but not with EB1 (Fig. 5*C*). Using similar coexpression approach, we performed a reverse co-IP with Myc antibody and reprobing with GFP antibody to detect GFP-tagged CLIP-170, CLASP2, and EB1. The results confirmed association of ectopically expressed SOCS3 with CLIP-170 and CLASP2, but not with EB1 (Fig. 5*D*).

### Characterization of SOCS3 protein domains involved in SOCS3 interaction with CLIP-170 and CLASP2

**Figure 6:**
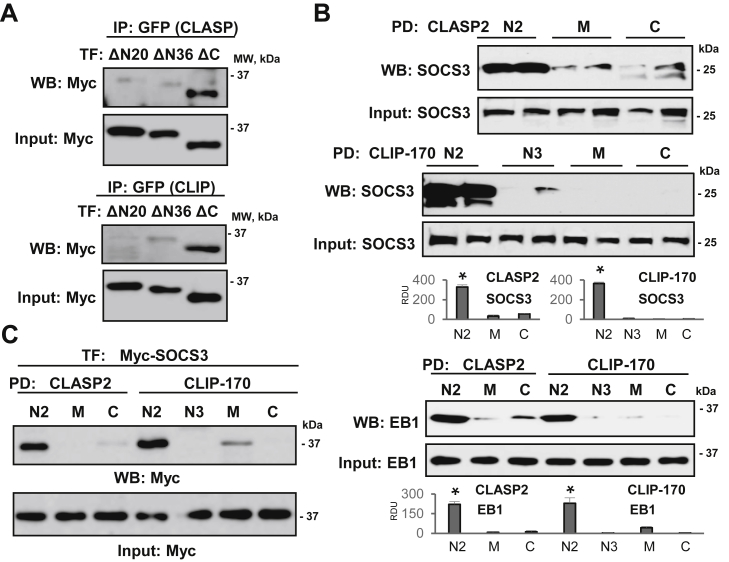
**N-terminal of SOCS3 is involved in its interaction with N2 domains of CLIP-170 and CLASP2**. *A*, ECs with coexpression of Myc-tagged N-terminal deletion mutants of SOCS3: ΔN20, ΔN36, or C-terminal SOCS3 deletion mutant (ΔC47) lacking SOCS box and GFP-CLASP2 (Top); or GFP-CLIP-170 (Bottom) were used for co-IP assay with GFP antibody followed by membrane probing with Myc antibody. Equal volumes of total cell lysates were probed with Myc antibody as normalization control. *B*, pull-down (PD) assay of EC lysates was performed with purified GST-fusion proteins encoding N2, M, and C domains of CLASP2 or N2, N3, M, and C domains of CLIP-170 (Top). Endogenous SOCS3 from cell lysates bound to immobilized CLASP2 and CLIP-170 domains was detected by western blot with SOCS3 antibody. Probing total cell lysates with SOCS3 antibody was used as a normalization control. Pull-down assay of End-Binding Protein 1 (EB1) (Bottom) served as a positive control to confirm published EB1 interaction with CLASP2 and CLIP-170 domains. *C*, pull-down experiment was carried out as in (*B*) after overexpression of SOCS3 followed by immunoblotting with Myc antibody. Bar graphs depict the results of quantitative densitometry of western blot data; n = 4, ∗*p* < 0.05.

To define the regions of SOCS3 interacting with MT-associated proteins, we carried out co-IP experiments using a set of SOCS3 deletion mutants. The results showed that SOCS3 mutants with deletion of the first 20 or 36 amino acids in N-terminal region, ΔN20 and ΔN36 respectively, failed to interact with CLIP-170 or CLASP2 (Fig. 6*A*). SOCS3 ΔC mutant that lacks the C-terminal region containing SOCS box was still able to associate with CLIP-170 or CLASP2. These data suggest that only N-terminal region of SOCS3 is involved in its interaction with the MT-associated proteins. Next, we proceeded to determine the regions of CLIP-170 and CLASP2 involved in the interaction with SOCS3. We performed pull-down assays with bacterially expressed and purified GST-fusion proteins containing various domains of CLIP-170 and CLASP2. We observed a strong interaction of SOCS3 with N2 domains of both CLIP-170 and CLASP2 (Fig. 6*B*, upper and middle panels). N2 domains of both CLIP-170 and CLASP2 were also involved in interaction with EB1 (Fig. 6*B*, lower panel). In consistence with the results obtained in experiments with endogenous proteins, the association of ectopically expressed SOCS3 with N2 domains of CLIP-170 and CLASP2 was also observed in pull-down experiments (Fig. 6*C*). Altogether, these data show that N-terminal region of SOCS3 interacts with N2 domains of CLIP-170 and CLASP2 in pulmonary endothelial cells.

### N-terminal region of SOCS3 is indispensable for its anti-inflammatory effects

**Figure 7:**
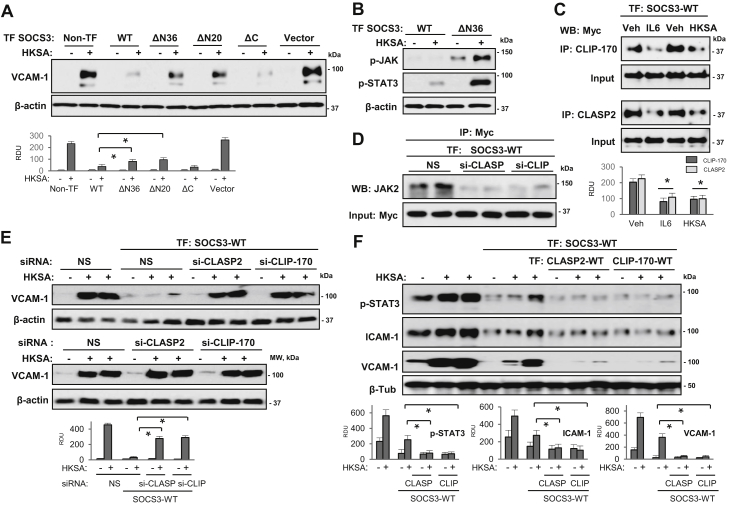
**N-terminal region of SOCS3 is essential for its inhibition of HKSA-induced inflammation**. *A*, pulmonary ECs were transfected with WT SOCS3 and indicated mutants or empty vector followed by HKSA stimulation (5 × 10^8^ particles/ml, 6 h) and western blot detection of VCAM-1 expression. Probing with β-actin was used as a normalization control. Bar graphs depict the results of quantitative densitometry of western blots; ∗*p* < 0.05, n = 5. *B*, phospho-JAK2 and phospho-STAT3 levels in HKSA-challenged EC with expression of Myc-SOCS3 or its Myc-ΔN36 mutant were detected by western blot. Probing with β-actin served as a normalization control. *C*, ECs with Myc-SOCS3 expression were exposed to IL-6 or HKSA (6 h), and co-IP with CLIP-170 and CLASP2 antibodies was followed by western blot with Myc antibody. *D*, ECs were cotransfected with nonspecific (NS), CLIP-170- or CLASP2-specific siRNAs and Myc-SOCS3 plasmid. Co-IP with Myc antibody was followed by western blot with JAK2 antibody. Probing of total cell lysates for Myc was used to verify equal inputs. *E*, ECs cotransfected with SOCS3-WT plasmid DNA and siRNAs targeting CLIP-170 or CLASP2 were treated with HKSA followed by western blot analysis of VCAM-1. Bar graph depicts the results of quantitative densitometry of western blots; n = 5, ∗*p* < 0.05. Lower panel: VCAM-1 expression in control and HKSA-stimulated EC transfected with nonspecific, CLIP-170-specific, or CLASP2-specific siRNAs; probing for β-actin served as a loading control. *F*, nontransfected pulmonary EC or cells with coexpression of SOCS3 and CLIP-170 or CLASP2 were stimulated with HKSA for indicated times. Phospho-STAT3, ICAM-1, and VCAM-1 expression in cell lysates was monitored by western blot. Bar graphs depict the results of quantitative densitometry of western blot data; n = 5, ∗*p* < 0.05. RDU, relative density units.

Since our new data provided the evidence that N-terminal region of SOCS3 is essential for its interaction with MT-associated proteins, we sought to determine the functional significance of this SOCS3 association in modulation of lung EC permeability and inflammatory response to HKSA. WT SOCS3 and its deletion mutants were ectopically expressed in pulmonary EC followed by HKSA stimulation, and VCAM-1 expression was monitored as readout of inflammatory activation. The results showed that only WT and ΔC SOCS3 mutant attenuated HKSA-induced VCAM-1 expression (Fig. 7*A*), while both ΔN SOCS3 mutants (lacking N-terminal 20 aa or 36 aa) were ineffective. These results suggest that the N-terminal region is absolutely essential for the anti-inflammatory function of SOCS3. Activation of the JAK-STAT pathway mediates the inflammatory signaling induced by IL-6 (35), and anti-inflammatory activities of SOCS3 had been credited to its capability to interfere with this pathway. Interestingly, overexpression of SOCS3 ΔN resulted in increase in basal as well as HKSA-induced phosphorylation of JAK2 and STAT3 (Fig. 7*B*), suggesting a requirement of N-terminal domain for restrictive effect of SOCS3 on HKSA-induced hyperactivation of JAK-STAT signaling and endothelial inflammation.

Since association of SOCS3 with the MT proteins appears to be critical for its anti-inflammatory functions, we next determined whether inflammatory agonists disrupt this interaction. Co-IP assays showed that both IL-6 and HKSA reduced SOCS3 interaction with CLIP-170 and CLASP2 (Fig. 7*C*). Furthermore, siRNA-induced knockdown of CLIP-170 or CLASP2 abolished the interaction of SOCS3 with JAK2 in pulmonary EC (Fig. 7*D*). We next determined effects of CLIP-170 or CLASP2 knockdown on SOCS3-mediated inhibition of inflammation. SOCS3 overexpression failed to rescue HKSA-induced upregulation of ICAM-1 expression in cells with depleted endogenous CLIP-170 or CLASP2 (Fig. 7*E*). These data indicate that particular MT end-binding proteins are required for the optimal SOCS3–JAK2 association in order to suppress inflammation. To further substantiate the role of SOCS3–CLIP-170/CLASP2 interactions in suppressing HKSA-induced endothelial inflammation, we coexpressed these proteins in pulmonary EC. The results showed that SOCS3 inhibitory effect on HKSA-induced ICAM-1 expression was further accentuated in EC with overexpressed CLIP-170 and CLASP2 (Fig. 7*F*).

### SOCS3/CLIP-170/CLASP2 complex is targeted by cytoskeletal scaffold protein IQGAP1

**Figure 8:**
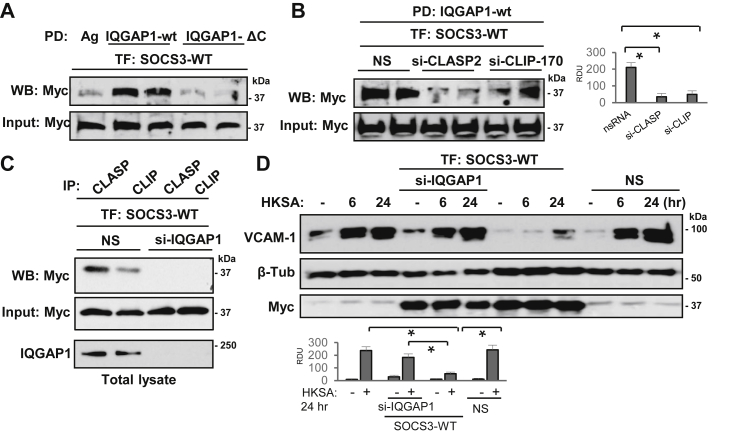
**SOCS3/CLIP-170/CLASP2 complex is targeted by cytoskeletal scaffold protein IQGAP1**. *A*, pull-down (PD) assay of Myc-SOCS3 using agarose beads with immobilized full-length IQGAP1 and its ΔC mutant. Bound SOCS3 fraction was detected by immunoblotting with Myc antibody. Probing of total lysates for Myc-SOCS3 was used as a normalization control. *B*, pull-down of SOCS3 on agarose beads with immobilized IQGAP1 from Myc-SOCS3-expressing EC with siRNA-depleted CLASP2 or CLIP-170. Probing of total lysates for Myc-SOCS3 was used as a normalization control. Bar graphs depict the results of quantitative densitometry of western blot data; n = 5, ∗*p* < 0.05. *C*, Myc-SOCS3-expressing ECs with siRNA-depleted IQGAP1 or cells treated with nonspecific (NS) RNA were tested in co-IP assays with CLASP2 or CLIP-170 antibodies. Presence of SOCS3 in immunocomplexes was monitored by western blot with Myc antibody. IQGAP1 depletion was confirmed by western blot. *D*, ECs were transfected with nonspecific or IQGAP1-specific siRNA, and ectopic expression of Myc-SOCS3 was performed. VCAM1 expression after HKSA challenge EC was determined by western blot. Control probing for β-tubulin and Myc-tag served as normalization and SOCS1 ectopic expression controls, respectively. Bar graphs depict the results of quantitative densitometry of western blot data; n = 4, ∗*p* < 0.05.

In the effort to clarify how MT-delivered SOCS3 signaling complex is anchored and stabilized at the submembrane region to exert sustained anti-inflammatory activity, we evaluated the role of IQGAP1, a multifunctional protein that is also involved in peripheral cytoskeletal remodeling and cell junction assembly. IQGAP1 also interacts with several MT proteins including CLIP-170 and CLASP2 and plays a role in linking MT to actin cytoskeleton (36). We investigated whether IQGAP1 is capable of recruiting SOCS3 and if this interaction also involves MT-binding proteins CLIP-170 and CLASP2. Pull-down assay with GST-IQGAP1 beads showed a firm association of SOCS3 with full-length IQGAP1 but not with ΔC truncated IQGAP1mutant that lacks C-terminal conserved region (Fig. 8*A*). Interestingly, the interaction of SOCS3 with full-length IQGAP was dramatically decreased after siRNA-mediated knockdown of CLIP-170 or CLASP2 (Fig. 8*B*). In turn, silencing of IQGAP1 abolished the interaction of SOCS3 with CLIP-170 and CLASP2 (Fig. 8*C*). Finally, IQGAP1 knockdown abolished anti-inflammatory effect of overexpressed SOCS3 monitored by induction of VCAM-1 levels in pulmonary EC challenged with HKSA (Fig. 8*D*). Taken together, these results strongly suggest that interaction of IQGAP1 with the SOCS3/CLIP-170/CLASP2 protein complex is essential for displaying full SOCS3 anti-inflammatory activity.

## Discussion

An important role of MT dynamics in regulating endothelial permeability and inflammation has been established by multiple studies including those from our group (29, 32, 37, 38, 39). SOCS3 has been described as a negative regulator of inflammation in some immune cells (28, 40), but whether it modulates inflammatory dysfunction in pulmonary endothelial cells was not investigated. This study tested the hypothesis that SOCS3 inhibits inflammatory signaling and protects barrier function in EC challenged with HKSA or IL-6, and SOCS3 function in pulmonary endothelium is MT-dependent. The data presented here suggest that direct association of SOCS3 with MT plus-end-binding proteins CLIP-170 and CLASP2 is required for full functionality of SOCS3 in inhibiting the HKSA- or IL-6-induced permeability and inflammation in pulmonary endothelial cells. These findings support the notion that proper intracellular positioning of SOCS3 by MT is essential for its anti-inflammatory effects in lung vasculature.

A recent study had shown that myeloid cell-specific knockout of SOCS3 in mice exacerbates LPS-induced ALI with activation of STAT3 and increased production of TNF-α (41). Another study showed that overexpression of SOCS3 protects against hyperglycemia-induced epithelial cell injury by inhibition of the JAK2-STAT3 pathway (42). Given that increased inflammatory response in EC is the major trigger for ALI, we were interested to determine if SOCS3 modulates endothelial dysfunction caused by inflammatory agents. Our results establish that SOCS3 plays a critical role in protecting against inflammatory ALI since siRNA-mediated depletion of endogenous SOCS3 in human lung EC and EC-specific SOCS3 knockout in mice exacerbated HKSA- or IL-6-induced lung injury. More importantly, SOCS3 overexpression rescued HKSA- or IL-6-induced endothelial hyperpermeability and inflammatory responses, suggesting that SOCS3 functions as a potent barrier protective and anti-inflammatory molecule in pulmonary EC. Protective effects of SOCS3 in sterile, IL-6-induced model of ALI described in this study may explain SOCS3-mediated protection of mechanical ventilation-induced lung injury shown in earlier reports (43, 44).

We have previously demonstrated that destabilization of MT mediates EC dysfunction caused by *Staphylococcus aureus* or its cell wall component peptidoglycan-G (32, 45). In agreement with these findings, this study demonstrates the requirement of intact MT for SOCS3-mediated protection of endothelial function against HKSA. The robust presence of SOCS3 in the MT fractions and loss of SOCS3-mediated protection of EC barrier function after inhibition of MT growth by low-dose nocodazole strongly support the MT-dependent mechanism of positive regulation of endothelial function by SOCS3. Our further experiments revealed that SOCS3 interacts with MT plus-end-binding proteins CLIP-170 and CLASP2, but not with EB1. MT plus-end-tracking proteins play an important role in stabilization of MT, and our previous studies have shown that CLASP2 is involved in strengthening of EC adherens junctions *via* its interaction with p120-catenin and regulation of VE-cadherin membrane localization (29). Corroborating with these published results, the data herein indicate that an association of SOCS3 with these MT proteins is essential for its positive regulation of endothelial function. This idea is further supported by the results that IL-6 or HKSA treatments disrupted the interaction of SOCS3 with CLIP-170 or CLASP2 in pulmonary EC (Fig. 7*C*). Moreover, depletion of CLIP-170 or CLASP2 impaired the interaction of SOCS3 with JAK2, indicating that these MT-associated proteins are essential for the inhibition of the JAK-STAT pathway by SOCS3.

Our *in vitro* binding assays showed that N2 domains of CLIP-170 and CLASP2 are involved in their association with SOCS3. It has been shown that N-terminal region of CLIP-170 containing MT binding domains is responsible for its interaction with IQGAP1 (46). Likewise, N-terminal region of CLASP2 rich in Serine/Arginine is involved in its association with IQGAP1 (47). In our studies, both endogenous and ectopically expressed SOCS3 showed a strong association with N2 domains of CLIP-170 and CLASP2. Multiple domains located at N-terminal of CLIP-170 are known as EB1 binding domains (48), and our results confirmed that EB1 binds to N2 domains of both CLIP-170 and CLASP2. However, SOCS3 did not bind directly to EB1, suggesting that among MT plus-end tracking proteins, only CLIP-170 and CLASP2 are crucial mediators of SOCS3 association with the MT.

Interestingly, SOCS3 interacted with IQGAP1 in a CLIP-170/CLASP2-dependent manner: SOCS3 did not coimmunoprecipitate with truncated IQGAP1 missing the C-terminal domain shown to interact with CLIP-170 and CLASP2 (46, 47). These results suggest that a large protein complex comprising CLIP-170, CLASP2, and IQGAP1 may be required for optimal targeting of SOCS3 to the submembrane compartment. We have previously shown that IQGAP1 is involved in MT–actin cross talk *via* its association with EB1 and mediates hepatocyte growth-factor-induced upregulation of endothelial barrier function (49). The present study uncovered the new important role of IQGAP1 in regulating SOCS3-mediated anti-inflammatory activity in pulmonary endothelium by modulating the interaction of SOCS3 with MT end-binding proteins. Further studies are warranted to reveal precise mechanism of this complex regulation and how such mechanism modulates SOCS3 anti-inflammatory activity in lung endothelium *in vivo*.

The inhibition of inflammatory signaling by SOCS3 is mediated either by its N-terminal KIR-mediated direct binding to JAK or by its C-terminal SOCS box-ubiquitin-mediated degradation of signaling complex (2, 11, 13). Our results strongly indicated that N-terminal region of SOCS3 is essential for its regulation of endothelial function as illustrated by the lack of interaction of SOCS3 ΔN (20 or 36 aa deletion) mutants with CLIP-170 and CLASP2. These interaction-defective ΔN SOCS3 mutants also failed to rescue HKSA-induced ICAM-1 expression, indicating the functional importance of N-terminal region of SOCS3. A more direct evidence of the functional significance of N-terminal domain was observed when ectopic expression of ΔN SOCS3 mutant caused a robust increase in the levels of phosphorylated JAK2 and STAT3 in unstimulated EC and further increased them in HKSA-challenged EC. However, SOCS3 ΔC mutant was as effective as WT in repressing HKSA-induced ICAM-1 expression, ruling out the functional significance of C-terminal region of SOCS3. These data are consistent with the notion that SOCS3 binding to JAK *via* its N-terminal is sufficient for the inhibition of JAK-STAT-mediated inflammatory signaling. However, SOCS box may play a role in some other modes of inflammatory diseases as reported previously (20).

Based on the presented data, we propose a mechanism of MT-dependent facilitation of SOCS3 anti-inflammatory activity. SOCS3 binds to CLASP2 and CLIP-170 N-terminal domains *via* its own 20-residue N-terminal domain and becomes loaded to and delivered by growing MT to the cell submembrane compartment, where N domains of CLASP2 and CLIP-170 associate with C-terminal domain of subcortical scaffold protein IQGAP1, and SOCS3 becomes anchored to the submembrane compartment where it exerts its anti-inflammatory activities.

In conclusion, the present study identifies a novel role of the MT in regulation of SOCS3-mediated protection of endothelial function against inflammatory agents. Our results show that SOCS3 association with MT plus-binding proteins drives its endothelial barrier protective and anti-inflammatory effects. These findings underscore the importance of MT stabilization and MT-associated proteins in preserving endothelial function.

## Experimental procedures

### Cell culture and reagents

Human pulmonary artery endothelial cells (HPAEC) and EGM-2 growth media kit were obtained from Lonza. Cells were used at passages 5–8, and all stimulations were done in media containing 2% fetal bovine serum. HKSA was purchased from InvivoGen; antibodies to ICAM1, VCAM1, GFP, CLIP-170, TLR4, and Myc were obtained from Santa Cruz Biotechnology; CLASP2 antibody was received from Millipore Sigma; β-actin and α- and β-tubulin antibodies were from Sigma; phospho-NFκB and SOCS3 antibodies were from Cell Signaling; EB1 antibody was obtained from BD Transduction Laboratories. Texas Red phalloidin and Alexa Flour 488 conjugated secondary antibodies were purchased from Molecular Probes. All other biochemical reagents were obtained from Sigma unless otherwise specified.

### DNA and siRNA transfections

DNA transfections were done using Lipofectamine LTX with Plus reagent from Thermo Fisher Scientific following manufacturer's instructions. After 24 h of transfections, cells were subjected for various assays or harvested for western blot analysis. Predesigned SOCS3, CLIP-170, CLASP2, and IQGAP1 siRNAs were obtained from Dharmacon and transfections were done using RNAiMAX reagent from Thermo Fisher Scientific. After 72 h posttransfections, cells were either harvested for western blot analysis or used for other assays. IQGAP1, CLIP-170 (46), CLASP2 (47) plasmids, and SOCS3 constructs (50) have been described previously.

### Measurement of endothelial permeability

The permeability across HPAEC monolayers was assessed by measuring transendothelial electrical resistance (TER) as well as by express permeability testing (XPerT) assay. TER was monitored over indicated time periods with an electrical cell–substrate impedance sensing system ECIS Z (Applied Biophysics) as described previously (51). EC permeability to macromolecules was evaluated by XPerT assay (52). Briefly, cells were grown on biotinylated gelatin-coated plates and at the end point of the experiments, FITC-avidin (25 μg/ml) was directly added to cells and incubated for 3 min. The unbound excess FITC was washed with PBS, and coverslips were mounted on slides with DAPI-containing mounting solution. The images were captured in a Nikon Eclipse TE300 inverted microscope.

### Coimmunoprecipitation

Cells were lysed on ice-cold TBS-NP40 lysis buffer (20 mM Tris pH 7.4, 150 mM NaCl, 1% NP40) supplemented with protease and phosphatase inhibitor cocktails (Roche). The clarified lysates were incubated with desired antibodies overnight at 4 °C followed by incubation with Protein G agarose beads for 1 h. After washing three times with TBS-NP40 lysis buffer, the immunocomplexes were eluted with 2x Laemmli buffer by heating at 95 °C for 5 min and analyzed by SDS-PAGE followed by western blotting. The relative intensities of the protein bands were quantified by scanning densitometry.

### Pull-down assay

GST-tagged proteins in pGEX vector were expressed in BL21 *Escherichia coli* strain and purified using glutathione resin (Clontech). Cells were lysed in buffer containing 50 mM Tris-HCl, pH 7.5, 150 mM NaCl, 1.5 mM MgCl_2_, 1 mM EDTA, 1% Triton X-100, 10% glycerol, supplemented with protease and phosphatase inhibitor cocktail. The lysates were clarified by centrifugation (13000*g*, 5 min) and incubated with GST-fusion proteins for 2 h at 4 °C. After washing three times with lysis buffer, resin-bound proteins were eluted followed by SDS-PAGE and western blotting.

### MT fractionation

MT-enriched fractions from cells after stimulations were extracted as previously described (31). Briefly, after agonist stimulation, cells were incubated with extraction buffer containing 100 mM Pipes pH 6.75, 1 mM EGTA, 1 mM MgSO_4_, 0.5% NP-40, protease, and phosphatase inhibitor cocktail for 10 min at room temperature. The cytosolic fraction containing soluble tubulin was collected at room temperature by centrifugation at 12,000 rpm for 15 min. The attached cells containing polymerized MT were collected, dissolved in SDS-PAGE lysis buffer, and used for analysis of MT pool by SDS-PAGE followed by western blotting.

### Immunofluorescence and image analysis

Cells plated on glass cover slips were fixed in 3.7% formaldehyde for 10 min and permeabilized with 0.1% Triton X-100 in PBS for 30 min. After blocking with 2% BSA in PBS for 30 min, incubation with primary antibodies was carried out for 1 h at room temperature followed by staining with Alexa-488 or Alexa-544 conjugated secondary antibodies. Actin filaments were stained with Texas Red-conjugated phalloidin diluted in the blocking solution. The immunostained slides were analyzed using a Nikon Eclipse TE300 inverted microscope connected to a Spot RT monochrome digital camera and image processor (Diagnostic Instruments). The acquired images were processed with Adobe Photoshop. Quantification of HKSA-induced gap formation by EC monolayers was performed as described elsewhere (30, 53, 54). The gap formation in Texas Red-stained EC monolayers was expressed as a ratio of the gap area to the area of the whole image. EC permeability tested in XPerT assays was quantified by measuring total fluorescence intensity of microscopic fields. The values were statistically processed using Sigma Plot 7.1 (SPSS Science) software. For each experimental condition, at least ten microscopic fields in each independent experiment were analyzed.

### Animal studies

All the protocols involving animal care and treatment procedures were approved by the Institutional Animal Care & Use Committee of University of Maryland. C57/BL6 mice were obtained from Jackson Laboratories. EC-specific SOCS3 knockout mice were generated by cross-breeding of the endothelial VECad-Cre-ER^T2^ line (55) and SOCS3^flox/flox^ mice (56) in the C57B6 background, as we previously described (57). Both sets of mice were anesthetized with an intraperitoneal injection of ketamine (75 mg/kg) and xylazine (7.5 mg/kg). Then, sterile saline solution or HKSA (2 × 10^8^ bacterial cells/mouse) was given intratracheally. IL-6 was injected in mice intravenously in a single dose of 100 ng. BAL fluid was collected after intratracheal injection of 1 ml of sterile Hanks' balanced salt buffer, and total cells and protein content in BAL were determined as described previously (58). The vascular leak was analyzed by injecting Evans blue dye (30 mg/kg) into the external jugular vein 2 h before the end of the experiment as described earlier (58) and after perfusion, excised lungs were imaged with a Kodak digital camera.

### Statistical analysis

Results are expressed as means ± SD of four to eight independent experiments. Stimulated samples were compared with control groups by unpaired Student's *t*-test. For multiple groups comparison, we used one-way analysis of variance (ANOVA) followed by the post hoc Tukey test. *p* < 0.05 was considered statistically significant.

## Data availability

All data are contained within the article.

## Conflict of interest

The authors declare that they have no conflicts of interest with the contents of this article.
